# A2 adenosine receptor contributes to the development of cow’s milk protein allergy via regulating regulatory T cells

**DOI:** 10.22038/IJBMS.2021.57614.12812

**Published:** 2021-10

**Authors:** Chuan-Ying Li, Zhen Qin, Shao-Hua Mei, Yan Hu, Cheng Wu

**Affiliations:** 1 Department of Gastroenterology, Anhui Provincial Children’s Hospital (Children’s Hospital of Anhui Medical University), Wangjiang East Road No.39, Hefei, 230051, China

**Keywords:** Adenosine receptor, Allergic response, Cow’s milk protein allergy, Foxp3, Regulatory T cells

## Abstract

**Objective(s):**

A2 adenosine receptor (A2AR) is a novel promising target for the treatment of inflammatory and allergic diseases. However, its role in the development of cow’s milk protein allergy (CMPA) has not been elucidated. The present study was designed to investigate the function of A2AR in CMPA development.

**Materials and Methods:**

BALB/c mice were sensitized and challenged with ovalbumin (OVA) to induce allergic responses. The model was assessed by detecting allergic responses and plasma-specific IgE levels. The levels of A2AR were measured by PCR and flow cytometry. The subpopulation of Treg cells was analysed.

**Results:**

The mice sensitized and challenged with OVA showed classic allergic symptoms, such as acute allergic skin responses, increased anaphylactic shock symptom scores, and higher levels of total IgE, OVA-speciﬁc IgE, IgG1 and IgG2a. OVA-sensitized mice and CMPA patients showed decreased levels of A2AR and Treg cells. Interestingly, we observed a positive correlation between A2AR expression and Treg levels in CMPA patients. Further study showed that the A2AR agonist CGS21680 blocked OVA-induced allergic reactions, and the A2AR antagonist KW-6002 amplified allergic responses. Interestingly, CGS21680 not only activated the A2AR-mediated signalling pathway but also caused an increase in the population of Treg cells. In contrast, KW-6002 therapy decreased the levels of Tregs in allergic mice.

**Conclusion:**

A2AR expression is downregulated in CMPA. The A2AR-mediated pathway negatively regulates the development of CMPA, at least in part, by amplifying the differentiation of Tregs.

## Introduction

Food allergies are a growing health concern in the westernized world, with approximately 6% of children suffering from food allergies (1). Cow’s milk protein allergy (CMPA) is the main cause of food allergy in infants and younger children. CMPA is an adverse effect arising from a specific immune response that occurs reproducibly upon exposure to a protein present in milk. CMPA symptoms include anaphylaxis, enterocolitis syndrome, dysphagia, abdominal pain, constipation, respiratory distress, wheezing, atopic eczema and angioedema (2–3). Approximately 50% of children can tolerate CMPA by 5 years of age, increasing to 75% by their early teenage years (4). Nevertheless, some children still experience persistent allergic reactions. Although great progress has been made in the diagnosis and treatment of CMPA, there is increasing interest in developing suitable therapies for CMPA other than avoidance and developing approaches for early, accurate, and rapid diagnosis of CMPA. Therefore, it is urgent to find and explore potential biomarkers and therapeutic targets.

Adenosine is an endogenous autacoid whose biological effects are achieved by activation of its G protein-coupled receptors: A1 adenosine receptor (A1AR), A2AR, A2BR and A3AR. A1AR and A3AR signals are modulated through G_i_ and G_o_ members of the G protein family, while A2AR and A2BR couple with G_s_ proteins, thereby activating intracellular signalling and resulting in excitatory or inhibitory biological effects (5). A2AR is extensively distributed in organisms, with high expression in the basal ganglia, nerves, blood and immune cells (6). Numerous researchers have confirmed powerful biological effects of A2AR in key physiological processes, ranging from neuromodulation to immune regulation and from vascular function to metabolic control (7). Recently, A2AR has been implicated in the pathological process of allergic diseases. For example, A2AR deficiency leads to impaired tracheal relaxation in an allergic mouse model, and selective A2AR agonists exert anti-inflammatory effects on airway inflammation and airway hyperresponsiveness (8–9). However, little is known about A2AR’s biological effects in the development of CMPA. Therefore, the present study assessed the contribution of A2AR to the occurrence of CMPA.

## Materials and Methods

***Patients’ recruitment***

This study was approved by the ethics committee of the Anhui Provincial Children’s Hospital, and all procedures involving human participants were performed in accordance with the ethical standards described in the 1964 Declaration of Helsinki and its later amendments. Informed consent was obtained from participants included in the study. A total of 30 patients (19 boys and 11 girls, mean age: 3.06 years) were enrolled (from 2018-03 to 2019-06) in the study. The patients were diagnosed with CMPA based on a positive response to oral cow’s milk challenge as described previously (10). The exclusion criteria were those who had other chronic diseases or infections at the time of diagnosis. After treatment with extensively hydrolysed protein formula for more than three months, CMPA patients were determined to be recovery control based on disappeared allergic performance, including cutaneous, gastrointestinal, and respiratory symptoms combined. Briefly, the CMPA symptoms included general discomfort in the form of persistent distress or colic, gastrointestinal (frequent regurgitation, vomiting, diarrhoea, etc.), blood in stools, respiratory (runny nose, otitis media), chronic cough, wheezing (unrelated to infection) and dermatological (atopic dermatitis, angio-oedema, urticaria unrelated to acute infections, drug intake, etc.) manifestations. Peripheral blood was collected from CMPA patients (before treatment) and recovered controls (three months after treatment) for further use.

***Animal model and treatment***

A total of 48 female BALB/c mice, 5 weeks old, were purchased from Charles River and maintained in an environment at 24°C and 65% humidity with a 12h light-dark cycle. The study involving animal use was approved by the ethics committee of the Children’s Hospital of Anhui Medical University, and all procedures in the study involving animals were performed in accordance with ethical standards described in the guidelines of laboratory animal use and care of the European Community (EEC Directive of 1986; 86/609/EEC). To induce allergic reactions (11), the mice in the ovalbumin (OVA) group were sensitized with 40 µg of OVA plus aluminium hydroxide as an adjuvant by intraperitoneal injection (IP) on days 0, 7 and 14. One week (day 21) after the final sensitization, all mice received an intradermal (ID) injection of 10 µg OVA in 20 µl PBS in both ears pinnae to determine acute allergic symptoms. Mice subsequently received intragastric administration (IG) challenged (16 hr after the ID challenge) with 50 mg OVA in 0.5 ml PBS. At the same time, the mice in the sham control group only received saline solution (sodium chloride 0.9% w/v) in the same manner as those in the OVA group. To evaluate the role of A2AR in the development of CMPA, the mice were treated with the A2AR agonist CGS21680 (IP purity>98%, Med Chem Express, NJ, USA) at a dose of 0.25 mg/kg and A2AR antagonist KW-6002 (purity>98%, Med Chem Express, NJ, USA) at a dose of 10 mg/kg subcutaneously beginning from the first sensitization (day 0) to the end of study (day 21).

***Assessment of allergic responses***

Allergic responses were determined. Ear thickness was measured in duplicate for each ear before and 1 hr after ID injection with OVA using a digital micrometer (Mitutoyo, Kawasaki, Japan). The body temperature of animals was measured from the rectum at 0, 10, 20, 30, 40, 50, and 60 min after ID injection. Moreover, the severity of anaphylactic shock was measured and scored 30 min after the ID challenge with a validated scoring table (12–13). Anaphylactic shock-induced reduction in body temperature was measured at 30 min after id challenge using a rectal thermometer. All measurements were performed blinded.

***Measurements of OVA-speciﬁc IgE, IgA, total IgE, and OVA-speciﬁc IgG1 and IgG2a***

Blood samples were collected from mice after final IG challenge and centrifuged at 1000 g for 10 min to separate serum. Serum was stored at -20°C. The levels of OVA-specific IgE, IgA, IgG1, and IgG2a were assayed using OVA as the capture Ag and biotinylated anti-mouse IgG1, IgG2a, or IgE monoclonal-Ab (mAb, BD Biosciences; San Diego, USA) as the detection Ab. Total serum IgE levels were determined by ELISA using paired mAbs specific for standard mouse IgE (BD Biosciences; San Diego, USA).

***Measurement of Treg***

Twenty-four hr after IG OVA challenge, the mice were killed, and Treg levels were measured. Peripheral blood mononuclear cells (PBMCs) and mesenteric lymph nodes from animals were prepared as described previously (14). CD4^+ ^T cells were purified from samples using a CD4+ isolation kit according to the manufacturer’s instructions (Vancouver, BC, Canada). To detect regulatory T cells (Tregs), the cells were treated with ionomycin (1 mg/ml, Sigma-Aldrich, USA), phorbol ester (50 mg/ml, Sigma-Aldrich, USA) and brefeldin (1 mg/ml, Sigma-Aldrich, USA). Finally, the cells were stained for transcription factor forkhead/winged-helix family transcriptional repressor p3 (FoxP3, BD Biosciences, USA) and CD25 antibodies (BD Biosciences, San Diego, USA). The cells were analysed and counted by ﬂow cytometry (FACSCalibur; BD Bioscience, San Diego, USA), and the data were analysed using Cell Quest software (Becton Dickinson).

***Measurement of IL-10***

24 hr after IG OVA challenge, the mice were killed, and IL-10 concentration levels were measured. CD4^+ ^T cells purified from PBMCs and lymph nodes were cultured for 48 hr in growth medium consisting of RPMI-1640 (HyClone, USA) supplemented with 10% foetal bovine serum (Gibco, New York, USA) and 1% penicillin-streptomycin (Gibco, New York, USA) and maintained at 37°C and 5% CO_2_ in a humidiﬁed incubator. The level of IL-10 in the supernatant was detected by a commercial ELISA kit (R&D Systems, Minneapolis, USA).

***Measurement of A2AR***

24 hr after IG OVA challenge, the mice were killed, and A2AR levels on CD4+ T cells were measured and detected by flow cytometry analysis. Briefly, cells were directly stained with the A2AR antibody (BD Biosciences, USA) in stain buffer and finally washed with staining buffer to remove unbound antibodies. Cells were subjected to flow cytometry (FACSCalibur; BD Bioscience, USA) and electronically gated on live cells for analysis. Moreover, to detect the mRNA levels of A2AR, total RNA was extracted using a Qiagen RNeasy Mini Kit (Qiagen, CA, USA) according to the instructions provided by the manufacturer. The levels of A2AR were determined using quantitative RT-PCR (Applied Biosystems, 7900 Fast RT-PCR system). Specific primers for A2AR were as follows: Forward: 5′- GGCTATCTCTGACCAACA-3′, Reverse: 3′-TGGCTTGA CATC TCTAATCT-5′. Relative expression of A2AR genes normalized to GAPDH was determined by the delta-delta Ct method.

***Measurement of cAMP content and PKA activity***

The cells were harvested and centrifuged at 1000 rpm for 5 min and then lysed. The cAMP content was quantified using a cAMP assay kit (Abcam, Cambridge, UK) according to the manufacturer’s instructions. Moreover, PKA activity was measured using an ELISA kit (Enzo Life Sciences International, Inc., USA).

***Statistical analysis***

Statistical analysis was performed using SPSS 17.0 software (SPSS, Chicago, IL, USA). Data are expressed as the means± SD as indicated. Differences between groups were compared using one-way analysis of variance followed by the LSD test. The significance level was shown as *P* value <0.05.

## Results

***Treg and A2AR levels in CMPA patients***

**Figure 1:**
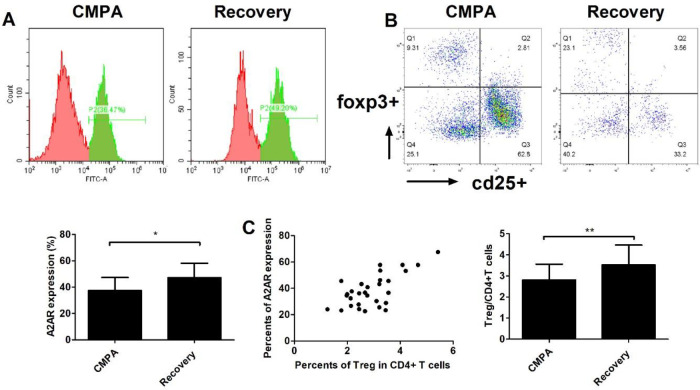
The levels of Treg and A2 adenosine receptor (A2AR) in cow’s milk protein allergy (CMPA) patients. A total of 30 CMPA patients were enrolled in the current study, of whom 13 presented gastrointestinal response, 6 presented cutaneous response, 5 presented respiratory symptoms, and the rest showed mixed symptoms. The patients received extensively hydrolysed protein formula instead of regular milk for three months, and all allergic responses disappeared in the enrolled patients. We defined them as recovery controls. Peripheral blood was collected from CMPA patients and recovery controls, and CD4+ T cells from peripheral blood were prepared. The levels of A2AR (A) and Treg (B) in CD4+ T cells were measured by flow cytometry analysis. Correlation analyses between A2AR and Tregs were carried out in a total of 30 CMPA patients using GraphPad Prism 5.0 software. Values are shown as the mean ± SD; n = 30; *P<0.05, **P<0.01 versus CMPA; ANOVA

To measure Treg and A2AR levels in CMPA patients, we enrolled a total of 30 CMPA patients in the study, in which 13 cases presented gastrointestinal responses, 6 cases presented cutaneous responses, 5 cases presented respiratory symptoms, and the remaining patients showed mixed symptoms. All patients received extensively hydrolysed protein formula instead of regular milk for three months, and all allergic responses disappeared in the enrolled patients. We defined them as recovery controls. As shown in Figure 1A-1B, the levels of A2AR and Treg cells were significantly lower in the CMPA status than in the recovery control status with the same clinical samples (*P*<0.01). Briefly, the expression of A2AR in CMPA patients and recovered controls was 37.4% and 47.3%, respectively. Moreover, the ratios of Tregs in the patients and recovered controls were 2.81% and 3.53%, respectively. A correlation analysis between the ratio of Tregs and the level of A2AR was conducted. Interestingly, we found a significant positive correlation (Pearson r: 0.6688; *P*<0.001) between the Treg and A2AR values in CMPA patients (Figure 1C, *P*<0.01).

***OVA sensitization induced allergic reactions and specific antibodies in a mouse model***

**Figure 2:**
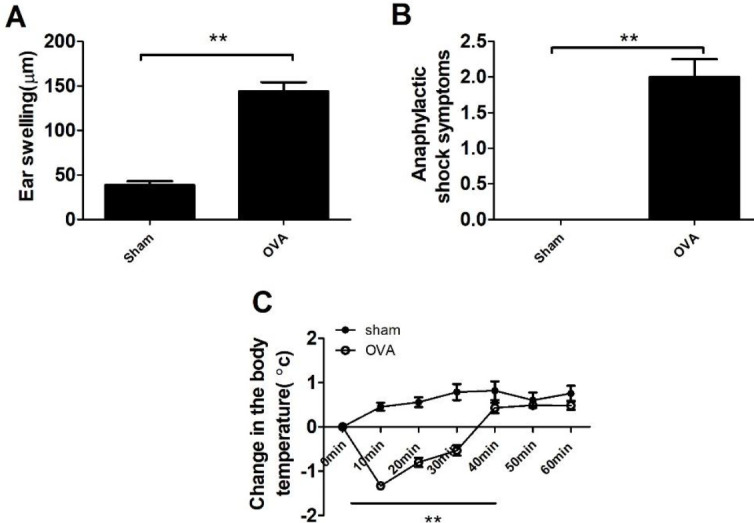
Allergic responses in ovalbumin (OVA)-treated mice. A: Ear swelling was measured before and 1 hr after intradermal injection (ID) challenge with OVA. A significant increase in ear swelling was observed in the OVA group. B: Anaphylactic shock reaction was scored before and 30 min after ID challenge. C: Anaphylactic shock-induced changes in body temperature were recorded before and 10–60 min after i.d. challenge. Data represent the means ± SD of individual mice (n=8) in the group. ** P<0.01, compared with sham group; ANOVA

**Figure 3:**
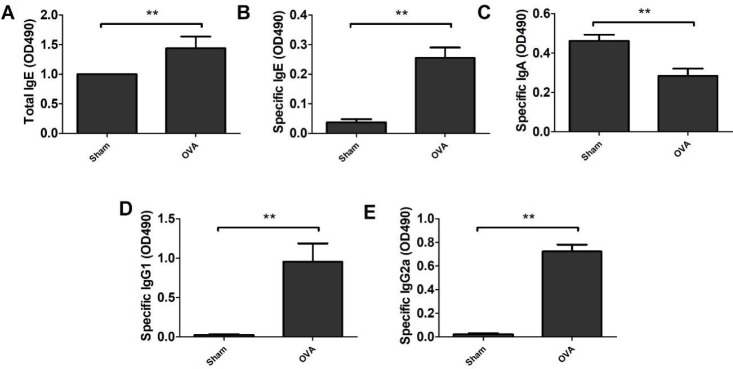
Specific antibodies in ovalbumin (OVA)-treated mice. The blood was collected from mice. Serum was separated, and the levels of total immunoglobulin E (IgE, A), OVA-speciﬁc IgE (B), speciﬁc IgA (C), speciﬁc IgG1 (D) and speciﬁc IgG2a (E) were quantified by ELISA kits. Data represent the means ± SD of individual mice (n=8) in the group. ** *P*<0.01, compared with sham group; ANOVA

Mice were orally sensitized by OVA several times and finally challenged to induce allergic reactions. As shown in Figure 2, there were significant increases in the acute allergic skin response and body temperature reductions in OVA-treated mice compared with sham control mice (*P*<0.01). Moreover, we measured the levels of specific immunoglobulins. As shown in Figure 3, mice treated with OVA showed significantly increased production of total IgE antibody, OVA-speciﬁc IgE, OVA-speciﬁc IgG1 and OVA-speciﬁc IgG2a as well as decreased production of speciﬁc IgA in the blood compared to sham control mice (*P*<0.01). Our data showed that the OVA-induced allergic model was established successfully.

***A2AR were downregulated in OVA induced allergic mice***

**Figure 4.**
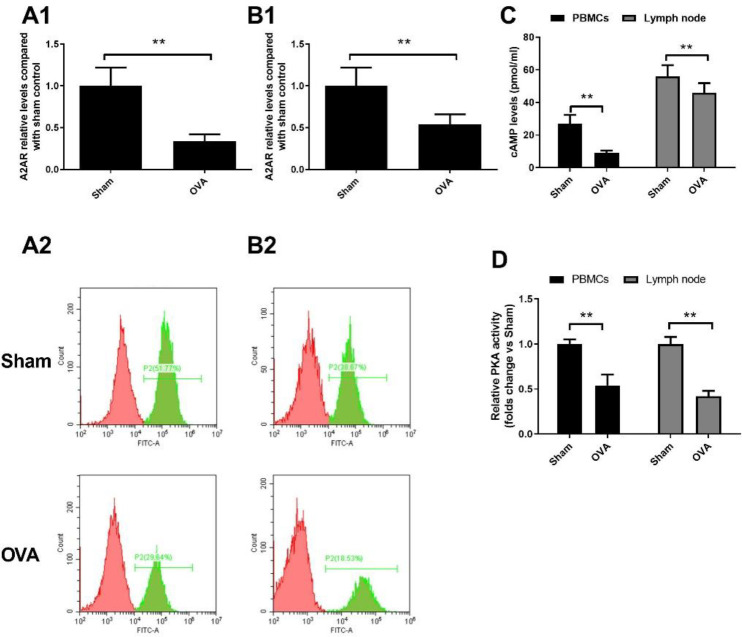
A2 adenosine receptor (A2AR)-mediated pathway in ovalbumin (OVA)-treated mice. CD4+ T cells from peripheral blood (A) and lymph nodes (B) were prepared. mRNA and expression of A2AR were measured by PCR and flow cytometry analysis. The content of the A2AR downstream molecule cAMP (C) and PKA activity (D) were assessed by a cAMP immunoassay kit and PKA kinase activity assay kit, respectively. Data represent the means±SD of individual mice (n=8) in the group. ** P<0.01, compared with sham group; ANOVA

It has been previously shown that A2AR and its mediated signalling pathway play an important role in allergic diseases (8–9). To determine the status of A2AR and its downstream signalling molecules, we separated CD4+ T cells and detected A2AR at the mRNA and protein levels by PCR and flow cytometry analysis, respectively. As presented in Figure 4A-4B, the mRNA level and expression of A2AR were obviously reduced in CD4+ T cells of OVA-treated mice compared with those of sham control mice (*P*<0.01). Moreover, the levels of A2AR in the lymph nodes of mice showed similar results. Afterwards, we detected downstream signalling molecules of A2AR. A significant reduction in cAMP contents and PKA activity was observed in OVA-treated mice compared to sham control mice (Figure 4C-4D, *P*<0.01). These data indicate that the A2AR-mediated signalling pathway was blocked in OVA-induced allergic mice.

***Treg were downregulated in OVA induced allergic mice***

**Figure 5:**
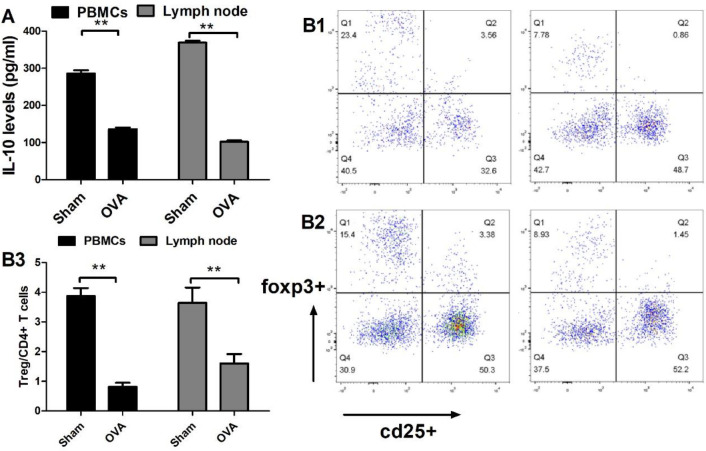
Regulatory T cell (Treg) levels in ovalbumin (OVA) treated mice. CD4+ T cells from peripheral blood (A) and lymph nodes (B) were prepared. The concentration of interleukin-10 (IL-10) was measured by ELISA, and the percentages of Tregs were measured by flow cytometry analysis. A: IL-10 level; B: B1 and B2 represent Tregs in peripheral blood mononuclear cells and lymph nodes, respectively, and B3 shows the quantified results. Data represent the means±SD of individual mice (n=8) in the group. ** *P*<0.01, compared with sham group; ANOVA

Tregs are functional immunosuppressive cells that induce immunological tolerance and play a negative role in the development of CMPA. It has been previously proven that A2AR regulates Treg differentiation (15). We therefore assessed the level of Tregs and their cytokine profile in allergic mice. Figure 5 shows that OVA-treated mice showed a 78.9% reduction in Tregs in peripheral CD4+ T cells and a 56.1% reduction in Tregs in lymphoid CD4+ T cells compared with control mice (*P*<0.01). Moreover, OVA-treated mice showed a 52.4% reduction in IL-10 in peripheral CD4+ T cells and a 72.4% reduction in Tregs in lymphoid CD4+ T cells compared with the control mice (*P*<0.01).

***A2AR agonist blocked OVA-induced allergic reaction, while A2AR antagonist reinforced it***

**Figure 6:**
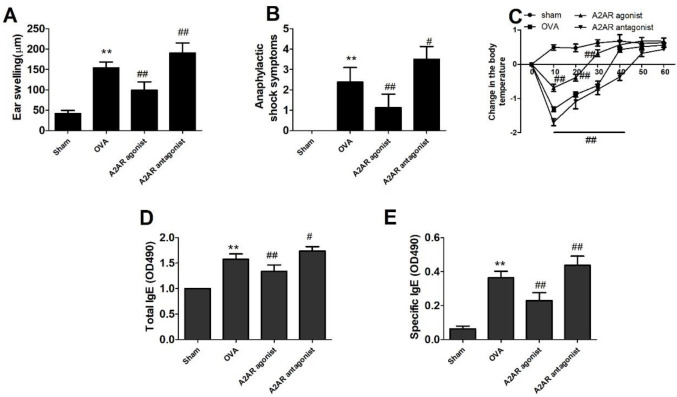
Effects of A2 adenosine receptor (A2AR) agonist and A2AR antagonist on ovalbumin (OVA)-induced allergic reaction in mice. Mice received the A2AR agonist CGS21680 and the A2AR antagonist KW-6002 daily 7 days before the final OVA challenge. A: Ear swelling was measured before and 1 hr after intradermal injection (ID) challenge with OVA. B: Anaphylactic shock reaction was scored before and 30 min after ID challenge. C: Anaphylactic shock-induced changes in body temperature were recorded before and 10–60 minutes after ID challenge. D: Immunoglobulin E (IgE); E: OVA-speciﬁc IgE. Data represent the means ± SD of individual mice (n=8) in the group. **P<0.01, compared with the sham group; #*P*<0.05, ##*P*<0.01, compared with the OVA group; ANOVA+LSD test

To determine the role of A2AR in CMPA, we used an A2AR agonist or antagonist to treat allergic mice. As shown in Fig. 6A-6C, treatment with the A2AR agonist CGS21680 reduced the severity of allergic reactions in mice by lowering ear swelling and systemic anaphylactic symptoms and decreasing body temperature (*P*<0.01). Conversely, we observed that the A2AR antagonist KW-6002 enhanced ear swelling and anaphylactic symptoms (*P*<0.01). Based on these results, we further investigated whether CGS21680 or KW-6002 could affect OVA-specific IgE levels. As shown in Figure 6D-6E, a significant decrease in the production of total IgE and OVA-specific IgE was observed in the serum of allergic mice treated with CGS21680 compared with that in OVA-treated control mice (*P*<0.01). In contrast, the mice that received KW-6002 exhibited increased levels of total IgE and OVA-specific IgE, as expected (*P*<0.01).

***A2AR agonists and antagonists regulate the levels of Treg cells***

**Figure 7:**
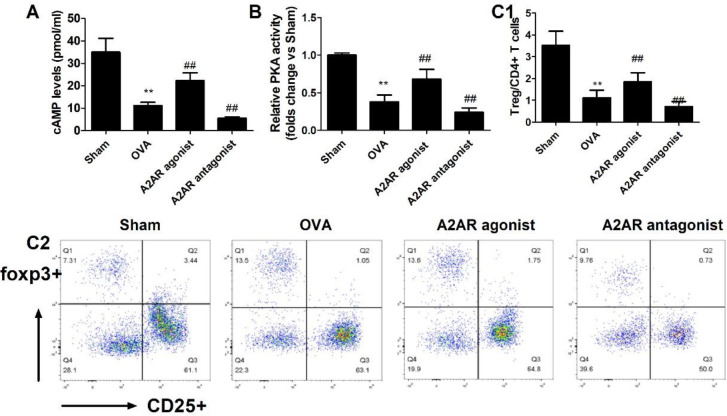
Effects of A2 adenosine receptor (A2AR) agonists and A2AR antagonists on the Treg population in allergic mice. Mice received CGS21680 and KW-6002 daily 7 days before the final ovalbumin (OVA) challenge, and peripheral CD4+ T cells were separated and purified. The content (A) and PKA activity (B) of the downstream molecules of cAMP were assessed by commercial analysis kits. The subpopulation of Tregs (C) in CD4+ T cells was detected by ﬂow cytometry analysis. Data represent the means ± SD of individual mice (n=8) in the group. **P< 0.01, compared with the sham group; #*P*<0.05, ##*P*<0.01 compared with the OVA group; ANOVA+LSD test

Treg negatively modulates CMPA. A2AR regulates Treg differentiation. To investigate whether Tregs contribute to the biological effects of A2AR, we further analysed the effects of A2AR agonists and A2AR antagonists on the production of Treg cells. As shown in Figure 7A-7B, CGS21680 and KW-6002 treatment for seven days effectively activated or blocked the A2AR-cAMP-PKA pathway, which was shown by alterations in the content of cAMP as well as the activity of PKA. Further investigation observed that CGS21680 caused a 65.1% increase in Treg levels, while KW-6002 caused a 45.7% decrease in Treg levels in OVA-treated mice (*P*<0.01). These data imply that Tregs contribute to the regulatory effects of A2AR on the OVA-induced allergic response.

## Discussion

In the present study, we aimed to determine the role of A2AR in the pathophysiology of CMPA. To achieve this goal, we established an allergic model in mice induced by OVA sensitization and challenge. Coadministration of OVA together with Th2 skewing agents such as aluminum hydroxide could induce classic isomorphic food allergic responses in animals, and these models help us understand the immune response and the transcriptional, cellular, and humoral profiles of the effector phase of food allergies, which are widely observed and demonstrated in human allergic responses (16-17). Therefore, the model has been widely used to study the pathological mechanism of milk protein-induced allergic responses. In our study, after challenge with OVA, a significant increase in ear swelling and hypothermia was observed in the OVA-treated mice. Further analysis showed that mice that received OVA and aluminum hydroxide displayed significantly higher levels of IgE and OVA-specific IgE, indicating an IgE-mediated allergic response in the mice. These symptoms together with increased levels of OVA-specific antibody levels indicate that a validated CMPA mouse model has been established.

The adenosine receptor has four subtypes, named A1AR, A2AR, A2BR and A3AR, based on the order of discovery. It was of interest to find that A1AR and A2AR are high affinity receptors combined with adenosine in the low to mid-nanomolar range, whereas A2BR has a substantially lower affinity for adenosine (micromolar) (18). Moreover, A2AR is extensively distributed in nerves, blood and immune cells. Previous research concluded that A2AR, but not A1AR or A3AR, was the predominant adenosine receptor on the surface of mouse T cells, and the expression and functional coupling of A2AR in mouse peripheral T cells and B cells correlated with adenosine-induced cAMP accumulation in lymphocytes (18–20). As demonstrated in a previous report, activation of A2AR blocks the effector functions of different subsets of mouse T cells (21). Therefore, A2AR is regarded as an important regulator of physiological and pathological processes connected with inflammation and the immune response. Recently, it was interesting to identify a key role of A2AR in the development of allergic disease. Allergen challenge resulted in a downregulatory effect on A2AR expression in asthma patients (22). Activation of A2AR by the agonist CGS-21680 prevented allergic responses and reduced airway hyperresponsiveness in mice during sensitization or rechallenge, which could be eliminated by myeloid-selective A2AR deletion (23). However, despite the proven efficacy of A2AR in the management of asthma, extensive concerns have arisen regarding its function in CMPA. Therefore, in the present study, we focused on A2AR expression on CD4+ T cells from peripheral blood and lymph nodes in a CMPA animal model. As observed, oral OVA sensitization and challenge led to decreased mRNA (66% reduction) and protein expression of A2AR in CD4+ T cells from peripheral blood and a remarkable reduction in mRNA (45% reduction) and protein expression of A2AR in CD4+ T cells from lymph nodes. We also found decreased expression of A2AR in CMPA patients compared with recovered controls. All these findings indicate that CMPA status downregulated A2AR in CD4+ T cells at both the gene and protein levels. A2AR couples with the GαS protein (18), which triggers increases in cellular cAMP content and PKA activity. To further determine the status of A2AR-mediated signalling in CMPA, we measured the content of cAMP and the activity of PKA using commercial ELISA kits and observed a reduction in cAMP levels and PKA activity. Further work showed that A2AR activation by CGS21680 blocked the OVA-induced allergic reaction, while KW-6002 reinforced it. These results are interesting and remind us that blockade of A2AR may result in temporarily predominant functions of A1AR and A2BR. In a previous study, A2BR stimulation was shown to promote Th17 differentiation by stimulating IL-6 expression in a cAMP-independent manner (18). Therefore, it needs to be elucidated whether A2AR contributes to the development of CMPA in our next study.

How does A2AR act as a negative regulator of CMPA? Treg cells are a subtype of CD4+ T cells that have a powerful ability to maintain immunological self-tolerance and suppress the development of various autoimmune diseases (24). The inhibitory function of Tregs on the immune response is closely associated with their direct connection with immune cells and secretion of the anti-inflammatory cytokine IL-10 (24). It is well accepted that CMPA is caused by the failure to develop and sustain oral immune tolerance to allergens, including aeroallergens and foods. An effective tolerogenic immune response towards allergens is a key factor in preventing the occurrence of allergic diseases (25). Tregs impair inflammatory properties by developing tolerogenic dendritic cell phenotypes, inhibiting mast cells, basophils and eosinophils directly, and blocking the influx of effector T cells into inflamed tissues in a cytokine-dependent manner (24). Previous investigations (26–28) have shown that the levels of Tregs are remarkably reduced in an allergic airway inflammation mouse model and allergic rhinitis in BALB/c mice and a CMPA mouse model. In contrast, it was interesting to see that supplementation of Tregs to CMPA rats could induce oral tolerance and inhibit the severity of the allergic response in CMPA mice (29). In the present study, CD4+ T cells were isolated from PBMCs and lymph nodes. We found that CD25+Foxp3+ T cells were significantly decreased in OVA-treated mice. Likewise, the Treg cell population was significantly decreased in CMPA patients. Afterwards, we confirmed that an A2AR agonist upregulated the Treg cell population in CMPA mice, while an A2AR antagonist exhibited the opposite effect. How did these results happen? Is there any relationship between the A2AR-mediated pathway and Treg cell differentiation? Masjedi *et al.* (15) found that the differentiation of conventional T cells purified from tumour-bearing mice to Tregs was blocked using A2AR-specific siRNA. Similarly, Wang *et al.* (30) found that Foxp3 (transcription factor of Treg) mRNA was positively correlated with A2AR mRNA in asthma patients. To support the above findings, we also confirmed that there was a significant positive correlation between the Treg and A2AR values in CMPA patients. Therefore, the regulatory effects of A2AR on Tregs (31), at least partially, are responsible for the observed negative effects of A2AR on CMPA.

## Conclusion

The expression of A2AR and its downstream molecules is downregulated in the development of CMPA. The A2AR-mediated pathway negatively regulates CMPA, at least in part, by amplifying the differentiation of Tregs. Our data indicate that A2AR may be a promising drug target for CMPA prevention.

## Authors’ Contributions

CYL and CW Study conception and design; CYL, ZQ, SHM and YH Data analyzing and draft manuscript preparation; CYL Critical revision of the paper; CW Supervision of the research; CYL, ZQ, SHM, YH and CW Final approval of the version to be published.

## Conflicts of Interest

The authors declare no conflict of interest.

## References

[1] Kostadinova AI, Willemsen LE, Knippels LM, Garssen J (2013). Immunotherapy-risk/benefit in food allergy. Pediatr Allergy Immunol.

[2] Vandenplas Y (2017). Prevention and management of cow’s milk allergy in non-exclusively breastfed infants. Nutrients.

[3] Hochwallner H, Schulmeister U, Swoboda I, Spitzauer S, Valenta R (2014). Cow’s milk allergy: From allergens to new forms of diagnosis, therapy and prevention. Methods.

[4] Taniuchi S, Takahashi M, Soejima K, Hatano Y, Minami H (2017). Immunotherapy for cow’s milk allergy. Hum Vaccin Immunother.

[5] Borea PA, Gessi S, Merighi S, Vincenzi F, Varani K (2018). Pharmacology of adenosine Receptors: The state of the art. Physiol Rev.

[6] Chen JF, Eltzschig HK, Fredholm BB (2013). Adenosine receptors as drug targets--what are the challenges?. Nat Rev Drug Discov.

[7] Al-Attraqchi OHA, Attimarad M, Venugopala KN, Nair A, Al-Attraqchi NHA (2019). Adenosine A2A receptor as a potential drug target - current status and future perspectives. Curr Pharm Des.

[8] Nadeem A, Ponnoth DS, Ansari HR, Batchelor TP, Dey RD, Ledent C (2009). A2A adenosine receptor deficiency leads to impaired tracheal relaxation via NADPH oxidase pathway in allergic mice. J Pharmacol Exp Ther.

[9] Caruso M, Varani K, Tringali G, Polosa R (2009). Adenosine and adenosine receptors: their contribution to airway inflammation and therapeutic potential in asthma. Curr Med Chem.

[10] Luyt D, Ball H, Makwana N, Green MR, Bravin K, Nasser SM (2014). Standards of care committee (socc) of the british society for allergy and clinical immunology (BSACI) BSACI guideline for the diagnosis and management of cow’s milk allergy. Clin Exp Allergy.

[11] Lebetwa N, Suzuki Y, Tanaka S, Nakamura S, Katayama S (2019). Enhanced anti-allergic activity of milk casein phosphopeptide by additional phosphorylation in ovalbumin-sensitized mice. Molecules.

[12] van Sadelhoff JHJ, Hogenkamp A, Wiertsema SP, Harthoorn LF, Loonstra R, Hartog A (2020). A free amino acid-based diet partially prevents symptoms of cow’s milk allergy in mice after oral sensitization with whey. Immun Inflamm Dis.

[13] Vonk MM, Blokhuis BRJ, Diks MAP, Wagenaar L, Smit JJ, Pieters RHH (2019). Butyrate enhances desensitization induced by oral immunotherapy in cow’s milk allergic mice. mediators inflamm.

[14] Song J, Xi JY, Yu WB, Yan C, Luo SS, Zhou L (2019). Inhibition of ROCK activity regulates the balance of Th1, Th17 and Treg cells in myasthenia gravis. Clin Immunol.

[15] Masjedi A, Hassannia H, Atyabi F, Rastegari A, Hojjat-Farsangi M, Namdar A (2019). Downregulation of A2AR by siRNA loaded PEG-chitosan-lactate nanoparticles restores the T cell mediated anti-tumor responses through blockage of PKA/CREB signaling pathway. Int J Biol Macromol.

[16] Kanagaratham C, Sallis BF, Fiebiger E (2018). Experimental models for studying food allergy. Cell Mol Gastroenterol Hepatol.

[17] Larsen JM, Bøgh KL (2018). Animal models of allergen-specific immunotherapy in food allergy: Overview and opportunities. Clin Exp Allergy.

[18] Cronstein BN, Sitkovsky M (2017). Adenosine and adenosine receptors in the pathogenesis and treatment of rheumatic diseases. Nat Rev Rheumatol.

[19] Huang S, Apasov S, Koshiba M, Sitkovsky M (1997). Role of A2a extracellular adenosine receptor- mediated signaling in adenosine-mediated inhibition of T-cell activation and expansion. Blood.

[20] Koshiba M, Kojima H, Huang S, Apasov S, Sitkovsky MV (1997). Memory of extracellular adenosine A2A purinergic receptor-mediated signaling in murine T cells. J Biol Chem.

[21] Lappas CM, Rieger JM, Linden J (2005). A2A adenosine receptor induction inhibits IFN-gamma production in murine CD4+ T cells. J Immunol.

[22] Versluis M, van den Berge M, Timens W, Luijk B, Rutgers B, Lammers JW (2008). Allergen inhalation decreases adenosine receptor expression in sputum and blood of asthma patients. Allergy.

[23] Pei H, Linden J (2016). Adenosine influences myeloid cells to inhibit aeroallergen sensitization. Am J Physiol Lung Cell Mol Physiol.

[24] Palomares O, Yaman G, Azkur AK, Akkoc T, Akdis M, Akdis CA (2010). Role of Treg in immune regulation of allergic diseases. Eur J Immunol.

[25] Kerperien J, Veening-Griffioen D, Wehkamp T, van Esch BCAM, Hofman GA, Cornelissen P (2018). IL-10 receptor or TGF-β neutralization abrogates the protective effect of a specific nondigestible oligosaccharide mixture in cow-milk-allergic mice. J Nutr.

[26] Hong JY, Kim M, Sol IS, Kim KW, Lee CM, Elias JA (2018). Chitotriosidase inhibits allergic asthmatic airways via regulation of TGF-β expression and Foxp3+ Treg cells. Allergy.

[27] Ren J, Zhao Y, Huang S, Lv D, Yang F, Lou L (2018). Immunomodulatory effect of bifidobacterium breve on experimental allergic rhinitis in BALB/c mice. Exp Ther Med.

[28] Adel-Patient K, Wavrin S, Bernard H, Meziti N, Ah-Leung S, Wal JM (2011). Oral tolerance and Treg cells are induced in BALB/c mice after gavage with bovine β-lactoglobulin. Allergy.

[29] Schouten B, van Esch BC, Hofman GA, Boon L, Knippels LM, Willemsen LE (2010). Oligosaccharide-induced whey-specific CD25(+) regulatory T-cells are involved in the suppression of cow milk allergy in mice. J Nutr.

[30] Wang L, Wan H, Tang W, Ni Y, Hou X, Pan L (2018). Critical roles of adenosine A2A receptor in regulating the balance of Treg/Th17 cells in allergic asthma. Clin Respir J.

[31] Ohta A, Kini R, Ohta A, Subramanian M, Madasu M, Sitkovsky M (2012). The development and immunosuppressive functions of CD4(+) CD25(+) FoxP3(+) regulatory T cells are under influence of the adenosine-A2A adenosine receptor pathway. Front Immunol.

